# Crystal structure of ethyl 2-[9-(5-bromo-2-hy­droxy­phen­yl)-1,8-dioxo-1,2,3,4,5,6,7,8,9,10-deca­hydro­acridin-10-yl]acetate

**DOI:** 10.1107/S2056989015022240

**Published:** 2015-11-25

**Authors:** Shaaban K. Mohamed, Mehmet Akkurt, Jerry P. Jasinski, Antar A. Abdelhamid, Asmaa H. Tamam, Mustafa R. Albayati

**Affiliations:** a Chemistry and Environmental Division, Manchester Metropolitan University, Manchester M1 5GD, England; b Chemistry Department, Faculty of Science, Minia University, 61519 El-Minia, Egypt; c Department of Physics, Faculty of Sciences, Erciyes University, 38039 Kayseri, Turkey; d Department of Chemistry, Keene State College, 229 Main Street, Keene, NH 03435-2001, USA; e Chemistry Department, Faculty of Science, Sohag University, 82524 Sohag, Egypt; f Kirkuk University, College of Science, Department of Chemistry, Kirkuk, Iraq

**Keywords:** crystal structure, acridines, hydro­acridinones, hydrogen bonding

## Abstract

In the title compound, C_23_H_24_BrNO_5_, the central 1,4-di­hydro­pyridine ring of the 1,2,3,4,5,6,7,8,9,10-deca­hydro­acridine ring system adopts a half-chair conformation. The two cyclo­hexene rings fused to the central ring both have a twisted-boat conformation. The mean planes of the bromo­hydroxy­phenyl ring and the major and minor components of the disordered ethyl amino­acetate moiety make dihedral angles of 78.99 (12), 85.9 (2) and 88.3 (9)°, respectively, with the 1,4-di­hydro­pyridine ring. The terminal ethyl group of the ethyl amino­acetate moiety is disordered over two sets of sites with refined occupancies of 0.768 (17) and 0.232 (17). The mol­ecular conformation is stabilized by an intra­molecular O—H⋯O hydrogen bond, forming an *S*(8) ring motif. In the crystal, C—H⋯O hydrogen bonds connect the mol­ecules into layers parallel to (001), enclosing *R*_1_^2^(7) ring motifs.

## Related literature   

For biological activities of hydro­quinolines, see: Moghadam *et al.* (2011 ▸); Miri *et al.* (2007 ▸). For accridinones, see: Okoro *et al.* (2012 ▸). For di­hydro­pyridines, see: Aydin *et al.* (2006 ▸); Rose (1990 ▸, 1991 ▸); Rose & Draeger (1992 ▸).
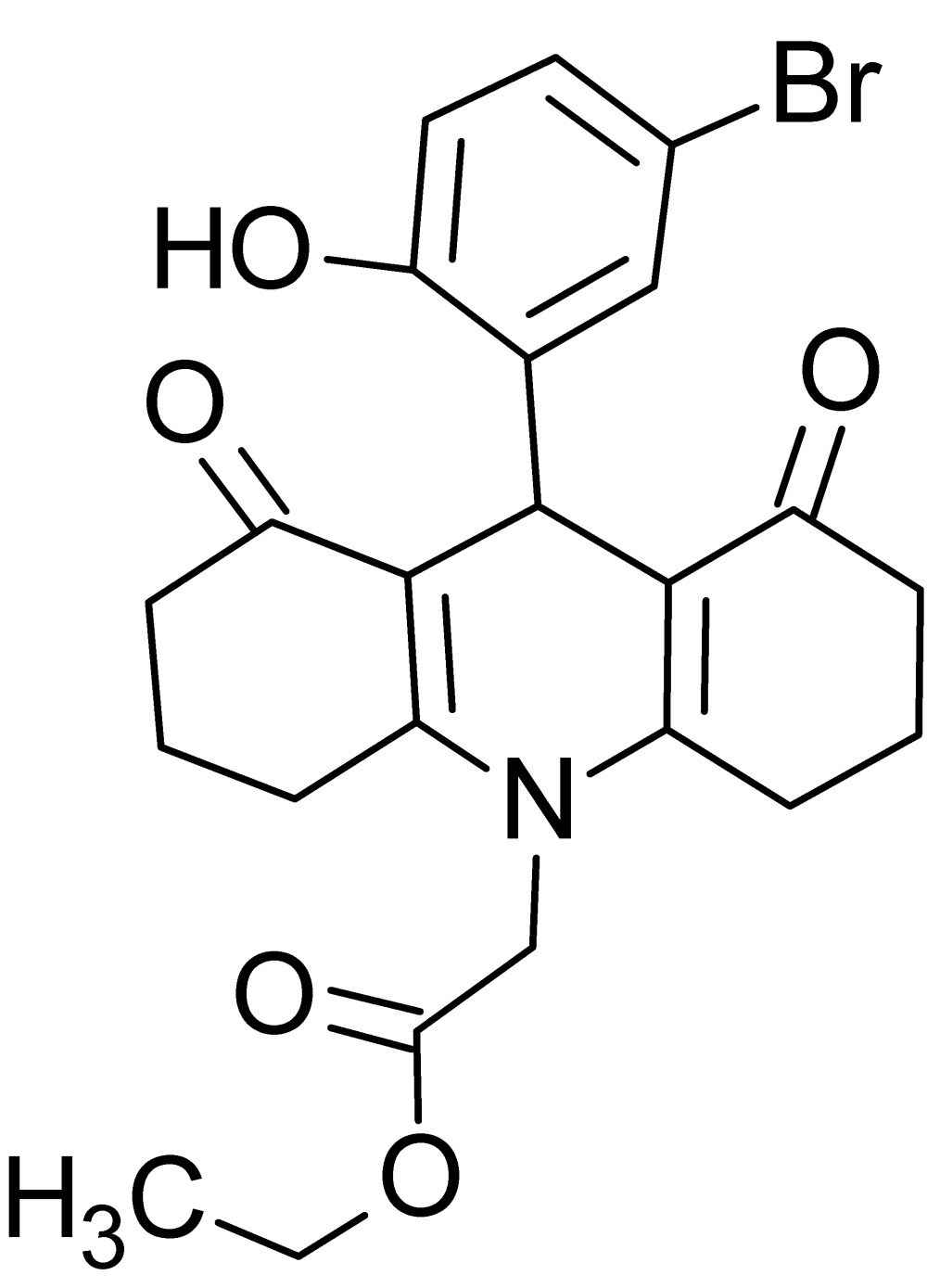


## Experimental   

### Crystal data  

C_23_H_24_BrNO_5_
*M* *_r_* = 474.33
Orthorhombic, 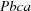

*a* = 8.8287 (3) Å
*b* = 14.2531 (5) Å
*c* = 33.1222 (11) Å
*V* = 4168.0 (2) Å^3^
*Z* = 8
Mo *K*α radiation
μ = 2.01 mm^−1^
*T* = 293 K
0.32 × 0.11 × 0.08 mm

### Data collection  

Agilent Xcalibur Eos Gemini diffractometer
Absorption correction: multi-scan (*CrysAlis PRO*; Agilent, 2014 ▸) *T*_min_ = 0.579, *T*_max_ = 1.000
51745 measured reflections
5281 independent reflections
3379 reflections with *I* > 2σ(*I*)
*R*_int_ = 0.060

### Refinement  

*R*[*F* ^2^ > 2σ(*F* ^2^)] = 0.059
*wR*(*F* ^2^) = 0.153
*S* = 1.02
5281 reflections
281 parameters
1 restraint
H atoms treated by a mixture of independent and constrained refinement
Δρ_max_ = 0.53 e Å^−3^
Δρ_min_ = −0.59 e Å^−3^

### 

Data collection: *CrysAlis PRO* (Agilent, 2014 ▸); cell refinement: *CrysAlis PRO*; data reduction: *CrysAlis PRO*; program(s) used to solve structure: *SHELXS2014* (Sheldrick, 2008 ▸); program(s) used to refine structure: *SHELXL2014* (Sheldrick, 2015 ▸); molecular graphics: *ORTEP-3 for Windows* (Farrugia, 2012 ▸); software used to prepare material for publication: *PLATON* (Spek, 2009 ▸) and *PARST* (Nardelli, 1995 ▸).

## Supplementary Material

Crystal structure: contains datablock(s) global, I. DOI: 10.1107/S2056989015022240/wm5239sup1.cif

Structure factors: contains datablock(s) I. DOI: 10.1107/S2056989015022240/wm5239Isup2.hkl

Supporting information file. DOI: 10.1107/S2056989015022240/wm5239Isup3.cml

**Fig. 1.**
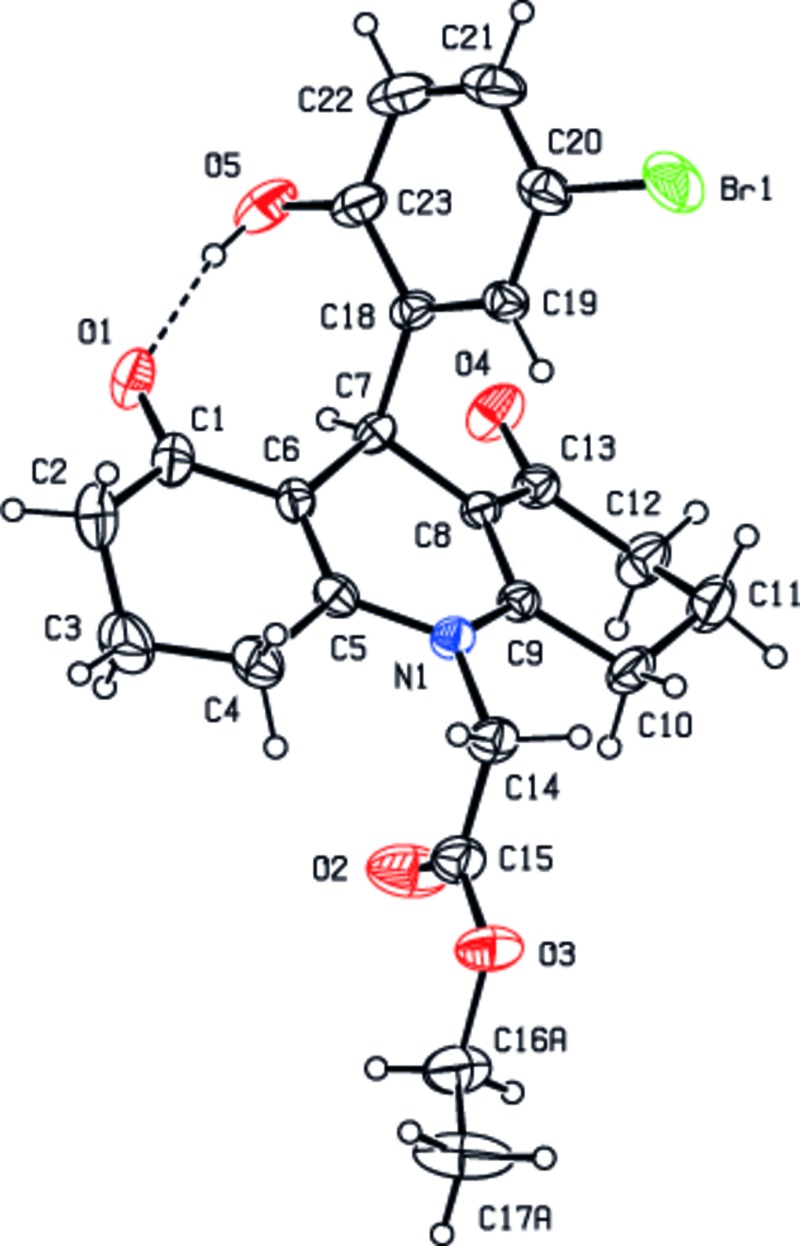
View of the title molecule with displacement ellipsoids drawn at the 30% probability level. Only the major component (C16A, C17A) of disorder is shown.

A A . DOI: 10.1107/S2056989015022240/wm5239fig1.tif
View of the title mol­ecule with displacement ellipsoids drawn at the 30% probability level. Only the major component (C16*A*, C17*A*) of disorder is shown.

**Fig. 2.**
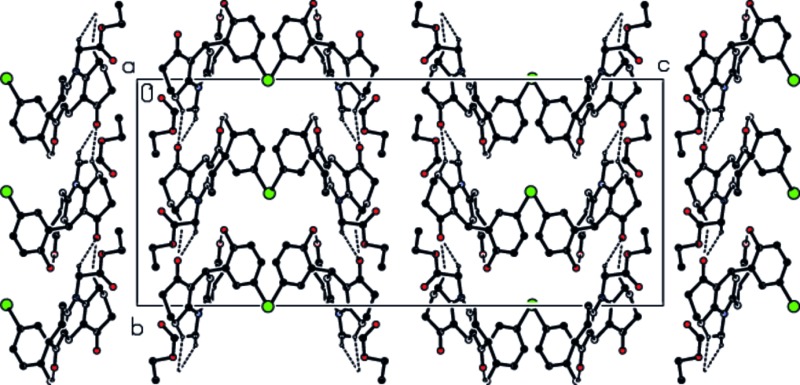
The packing of molecules in the the title compound viewed down [100]. Hydrogen bonds are shown as dashed lines. Hydrogen atoms not involved in hydrogen bonding and the minor component of disorder have been removed for clarity.

. DOI: 10.1107/S2056989015022240/wm5239fig2.tif
The packing of mol­ecules in the the title compound viewed down [100]. Hydrogen bonds are shown as dashed lines. Hydrogen atoms not involved in hydrogen bonding and the minor component of disorder have been removed for clarity.

CCDC reference: 1437968

Additional supporting information:  crystallographic information; 3D view; checkCIF report

## Figures and Tables

**Table 1:** Hydrogen-bond geometry (Å, °)

*D*—H⋯*A*	*D*—H	H⋯*A*	*D*⋯*A*	*D*—H⋯*A*
O5—H5⋯O1	0.83 (3)	1.91 (3)	2.709 (4)	163 (6)
C10—H10*A*⋯O4^i^	0.97	2.41	3.228 (4)	142
C14—H14*B*⋯O4^i^	0.97	2.42	3.267 (4)	146

Symmetry code: (i) 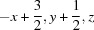.

## supplementary crystallographic information

### S1. Comment

Hydroquinolines (Moghadam *et al.*, 2011; Miri *et al.*, 2007), acridinediones (Okoro *et al.*, 2012) and other anellated dihydropyridines (Aydin *et al.*, 2006; Rose, 1990) were developed that exhibit selective cardial agonist activity while calcium antagonistic effects were observed on smooth musculature (Rose & Draeger, 1992; Rose, 1991). In this context, we report here the synthesis and crystal structure of the title compound, C_23_H_24_BrNO_5_, (I).

In the structure of (I) (Fig. 1), the central 1,4-dihydropyridine ring (N1/C5–C9) of the 1,2,3,4,5,6,7,8,9,10-decahydroacridine ring system (N1/C1–C13) adopts a half-chair conformation [the puckering parameters are Q_T_ = 0.235 (3) Å, θ = 102.0 (7) °, φ = 6.0 (7) °]. The two cyclohexene rings (C1–C6 and C8–C13) of the 1,2,3,4,5,6,7,8,9,10-decahydroacridine ring system have a twisted-boat conformation [the puckering parameters are Q_T_ = 0.428 (4) Å, θ = 51.0 (5) °, φ = 109.2 (6) °, and Q_T_ = 0.468 (4) Å, θ = 60.6 (4) °, φ = 187.1 (5) °, respectively].

The mean planes of the bromo-hydroxyphenyl ring (C18–C23) and the major and minor components of the disordered ethyl aminoacetate moiety make dihedral angles of 78.99 (12), 85.9 (2) and 88.3 (9)°, respectively, with the 1,4-dihydropyridine ring (N1/C5–C9).

All bond lengths and bond angles in the title molecule are within the normal ranges and comparable with each other and with those obtained earlier for similar compounds.

The molecular conformation is stabilized by an intramolecular O—H···O hydrogen bond, which generates an *S*(8) ring motif (Fig. 1, Table 1).

In the crystal, molecules are linked by intermolecular C—H···O hydrogen bonds into layers parallel to (001), enclosing *R*^2^_1_(7) ring motifs (Table 1, Fig. 2).

### S2. Experimental

A mixture of 1 mmol (112 mg) of cyclohexane-1,3-dione, 1 mmol (201 mg) of 4-bromo-2-hydroxybenzaldehyde and 1 mmol (103 mg) ethyl 2-aminoacetate in 30 ml ethanol was refluxed at 351 K. The reaction was monitored by TLC until completion. The solid product was deposited on cooling and collected by filtration under vacuum. Recystallization of the crude product from ethanol afforded crystals sufficient for X-ray diffraction. M.p. 515 K.

### S3. Refinement

The hydroxyl hydrogen atom was found from a difference Fourier map and its O—H bond length was restrained using a *DFIX* restraint of 0.82 (2) Å, with its displacement parameter set equal to 1.5*U*_eq_(O). The other H atoms were placed in calculated positions with C—H = 0.93 - 0.98 Å, and refined as riding with *U*_iso_(H) = 1.2*U*_eq_(C). The terminal ethyl group (C16, C17) of the ethyl aminoacetate moiety is disordered over two sets of sites with an occupancy ratio of 0.768 (17):0.232 (17).

### Crystal data

**Table:**

C_23_H_24_BrNO_5_	*F*(000) = 1952
*M**_r_* = 474.33	*D*_x_ = 1.512 Mg m^−^^3^
Orthorhombic, *P**b**c**a*	Mo *K*α radiation, λ = 0.71073 Å
Hall symbol: -P 2ac 2ab	Cell parameters from 4787 reflections
*a* = 8.8287 (3) Å	θ = 3.7–24.1°
*b* = 14.2531 (5) Å	µ = 2.01 mm^−^^1^
*c* = 33.1222 (11) Å	*T* = 293 K
*V* = 4168.0 (2) Å^3^	Needle, colourless
*Z* = 8	0.32 × 0.11 × 0.08 mm

### Data collection

**Table:**

Agilent Xcalibur Eos Gemini diffractometer	5281 independent reflections
Radiation source: Enhance (Mo) X-ray Source	3379 reflections with *I* > 2σ(*I*)
Graphite monochromator	*R*_int_ = 0.060
Detector resolution: 16.0416 pixels mm^-1^	θ_max_ = 28.5°, θ_min_ = 2.9°
ω scans	*h* = −11→11
Absorption correction: multi-scan (*CrysAlis PRO*; Agilent, 2014)	*k* = −19→19
*T*_min_ = 0.579, *T*_max_ = 1.000	*l* = −44→44
51745 measured reflections	

### Refinement

**Table:**

Refinement on *F*^2^	1 restraint
Least-squares matrix: full	Hydrogen site location: mixed
*R*[*F*^2^ > 2σ(*F*^2^)] = 0.059	H atoms treated by a mixture of independent and constrained refinement
*wR*(*F*^2^) = 0.153	*w* = 1/[σ^2^(*F*_o_^2^) + (0.052*P*)^2^ + 5.0593*P*] where *P* = (*F*_o_^2^ + 2*F*_c_^2^)/3
*S* = 1.02	(Δ/σ)_max_ = 0.001
5281 reflections	Δρ_max_ = 0.53 e Å^−^^3^
281 parameters	Δρ_min_ = −0.59 e Å^−^^3^

### Special details

**Table:**

Geometry. Bond distances, angles *etc*. have been calculated using the rounded fractional coordinates. All su's are estimated from the variances of the (full) variance-covariance matrix. The cell e.s.d.'s are taken into account in the estimation of distances, angles and torsion angles
Refinement. Refinement on *F*^2^ for ALL reflections except those flagged by the user for potential systematic errors. Weighted *R*-factors *wR* and all goodnesses of fit *S* are based on *F*^2^, conventional *R*-factors *R* are based on *F*, with *F* set to zero for negative *F*^2^. The observed criterion of *F*^2^ > σ(*F*^2^) is used only for calculating -*R*-factor-obs *etc*. and is not relevant to the choice of reflections for refinement. *R*-factors based on *F*^2^ are statistically about twice as large as those based on *F*, and *R*-factors based on ALL data will be even larger.

### Fractional atomic coordinates and isotropic or equivalent isotropic displacement parameters (Å^2^)

**Table:**

	*x*	*y*	*z*	*U*_iso_*/*U*_eq_	Occ. (<1)
Br1	0.93921 (6)	0.00364 (4)	0.25138 (2)	0.0856 (2)	
O1	0.3868 (3)	−0.27352 (16)	0.34249 (8)	0.0639 (9)	
O2	0.3448 (4)	0.0831 (2)	0.45391 (9)	0.0919 (13)	
O3	0.3033 (3)	0.22761 (18)	0.43222 (9)	0.0716 (10)	
O4	0.8428 (3)	−0.19850 (15)	0.42308 (8)	0.0626 (9)	
O5	0.6762 (3)	−0.33400 (18)	0.33476 (10)	0.0738 (10)	
N1	0.4997 (3)	0.03570 (15)	0.38366 (7)	0.0386 (7)	
C1	0.3472 (4)	−0.1908 (2)	0.34564 (10)	0.0487 (11)	
C2	0.1926 (4)	−0.1605 (3)	0.33252 (13)	0.0640 (11)	
C3	0.1358 (4)	−0.0829 (3)	0.35687 (14)	0.0781 (15)	
C4	0.2432 (4)	−0.0007 (2)	0.35809 (11)	0.0545 (11)	
C5	0.4030 (3)	−0.0314 (2)	0.36740 (9)	0.0382 (8)	
C6	0.4505 (3)	−0.12057 (19)	0.36125 (8)	0.0360 (8)	
C7	0.6109 (3)	−0.15066 (18)	0.36981 (8)	0.0347 (8)	
C8	0.6843 (3)	−0.08124 (18)	0.39812 (8)	0.0319 (8)	
C9	0.6337 (3)	0.00739 (18)	0.40212 (8)	0.0350 (8)	
C10	0.7157 (4)	0.0782 (2)	0.42760 (11)	0.0526 (11)	
C11	0.8757 (4)	0.0494 (2)	0.43624 (13)	0.0643 (14)	
C12	0.8835 (4)	−0.0486 (2)	0.45150 (11)	0.0590 (12)	
C13	0.8066 (3)	−0.1162 (2)	0.42346 (9)	0.0410 (9)	
C14	0.4438 (4)	0.1312 (2)	0.38959 (10)	0.0473 (10)	
C15	0.3597 (4)	0.1431 (2)	0.42924 (12)	0.0546 (11)	
C16A	0.2173 (11)	0.2445 (7)	0.4704 (2)	0.076 (3)	0.768 (17)
C16B	0.261 (4)	0.281 (2)	0.4685 (9)	0.076 (3)	0.232 (17)
C17A	0.1256 (11)	0.3290 (10)	0.4650 (2)	0.109 (4)	0.768 (17)
C17B	0.108 (5)	0.271 (3)	0.4674 (9)	0.109 (4)	0.232 (17)
C18	0.7040 (3)	−0.1655 (2)	0.33158 (9)	0.0389 (8)	
C19	0.7647 (3)	−0.0892 (2)	0.31119 (8)	0.0408 (9)	
C20	0.8555 (4)	−0.1024 (3)	0.27790 (10)	0.0544 (10)	
C21	0.8894 (4)	−0.1919 (4)	0.26418 (11)	0.0693 (14)	
C22	0.8278 (5)	−0.2668 (3)	0.28343 (12)	0.0690 (16)	
C23	0.7340 (4)	−0.2559 (2)	0.31685 (11)	0.0539 (11)	
H2A	0.19630	−0.14140	0.30440	0.0770*	
H2B	0.12350	−0.21310	0.33470	0.0770*	
H3A	0.11830	−0.10490	0.38420	0.0940*	
H3B	0.03940	−0.06220	0.34600	0.0940*	
H4A	0.20970	0.04340	0.37850	0.0650*	
H4B	0.24150	0.03120	0.33220	0.0650*	
H5	0.584 (3)	−0.327 (4)	0.3388 (18)	0.1280*	
H7	0.60600	−0.21100	0.38390	0.0420*	
H10A	0.71620	0.13820	0.41370	0.0630*	
H10B	0.66200	0.08620	0.45290	0.0630*	
H11A	0.93530	0.05460	0.41180	0.0770*	
H11B	0.91880	0.09150	0.45620	0.0770*	
H12A	0.83570	−0.05180	0.47780	0.0710*	
H12B	0.98880	−0.06660	0.45470	0.0710*	
H14A	0.37650	0.14730	0.36750	0.0570*	
H14B	0.52870	0.17430	0.38890	0.0570*	
H16B	0.15260	0.19120	0.47630	0.0910*	0.768 (17)
H16C	0.28690	0.25290	0.49280	0.0910*	0.768 (17)
H16D	0.30410	0.25330	0.49270	0.0910*	0.232 (17)
H16E	0.29200	0.34600	0.46650	0.0910*	0.232 (17)
H17A	0.06790	0.34060	0.48900	0.1640*	0.768 (17)
H17B	0.05800	0.32030	0.44260	0.1640*	0.768 (17)
H17C	0.19080	0.38150	0.45980	0.1640*	0.768 (17)
H17D	0.06370	0.30440	0.48980	0.1640*	0.232 (17)
H17E	0.08130	0.20620	0.46900	0.1640*	0.232 (17)
H17F	0.06960	0.29720	0.44260	0.1640*	0.232 (17)
H19	0.74390	−0.02870	0.32010	0.0490*	
H21	0.95330	−0.20040	0.24220	0.0830*	
H22	0.84880	−0.32690	0.27400	0.0830*	

### Atomic displacement parameters (Å^2^)

**Table:**

	*U*^11^	*U*^22^	*U*^33^	*U*^12^	*U*^13^	*U*^23^
Br1	0.0760 (3)	0.1199 (4)	0.0608 (3)	−0.0183 (3)	0.0258 (2)	0.0017 (2)
O1	0.0663 (16)	0.0464 (14)	0.0790 (18)	−0.0189 (12)	−0.0077 (13)	−0.0061 (12)
O2	0.128 (3)	0.0738 (19)	0.0739 (19)	0.0340 (19)	0.0361 (19)	0.0174 (16)
O3	0.0829 (19)	0.0565 (15)	0.0754 (18)	0.0255 (14)	0.0114 (14)	−0.0102 (13)
O4	0.0684 (16)	0.0387 (12)	0.0806 (17)	0.0162 (11)	−0.0263 (13)	−0.0053 (11)
O5	0.099 (2)	0.0375 (13)	0.085 (2)	0.0067 (15)	−0.0074 (18)	−0.0181 (13)
N1	0.0388 (12)	0.0304 (11)	0.0466 (14)	0.0060 (10)	−0.0021 (11)	−0.0012 (10)
C1	0.0490 (18)	0.0478 (19)	0.0494 (18)	−0.0132 (15)	−0.0033 (15)	0.0018 (14)
C2	0.0460 (19)	0.072 (2)	0.074 (2)	−0.0196 (18)	−0.0144 (18)	0.009 (2)
C3	0.0402 (19)	0.110 (3)	0.084 (3)	0.001 (2)	−0.011 (2)	−0.010 (3)
C4	0.0366 (15)	0.065 (2)	0.062 (2)	0.0090 (15)	−0.0047 (16)	0.0018 (17)
C5	0.0347 (14)	0.0435 (15)	0.0364 (14)	0.0027 (12)	0.0012 (12)	0.0044 (12)
C6	0.0340 (14)	0.0395 (15)	0.0346 (14)	−0.0068 (12)	0.0003 (11)	0.0021 (12)
C7	0.0421 (15)	0.0253 (12)	0.0366 (14)	0.0000 (11)	−0.0024 (12)	−0.0011 (11)
C8	0.0349 (13)	0.0286 (13)	0.0321 (13)	0.0009 (11)	0.0028 (11)	−0.0026 (10)
C9	0.0359 (13)	0.0326 (14)	0.0364 (13)	0.0009 (11)	0.0022 (11)	0.0002 (11)
C10	0.056 (2)	0.0357 (16)	0.066 (2)	0.0024 (14)	−0.0072 (17)	−0.0151 (15)
C11	0.058 (2)	0.052 (2)	0.083 (3)	−0.0059 (17)	−0.016 (2)	−0.0189 (19)
C12	0.060 (2)	0.055 (2)	0.062 (2)	0.0062 (17)	−0.0251 (18)	−0.0121 (17)
C13	0.0406 (15)	0.0386 (16)	0.0437 (16)	0.0049 (13)	−0.0027 (13)	−0.0018 (13)
C14	0.0518 (18)	0.0330 (15)	0.0571 (19)	0.0108 (14)	−0.0006 (15)	0.0009 (14)
C15	0.0530 (19)	0.0468 (19)	0.064 (2)	0.0106 (16)	0.0004 (17)	−0.0051 (17)
C16A	0.098 (6)	0.060 (5)	0.070 (3)	0.013 (4)	0.020 (3)	−0.007 (4)
C16B	0.098 (6)	0.060 (5)	0.070 (3)	0.013 (4)	0.020 (3)	−0.007 (4)
C17A	0.133 (6)	0.134 (9)	0.061 (3)	0.085 (7)	0.011 (3)	−0.005 (5)
C17B	0.133 (6)	0.134 (9)	0.061 (3)	0.085 (7)	0.011 (3)	−0.005 (5)
C18	0.0371 (15)	0.0423 (15)	0.0372 (14)	0.0068 (12)	−0.0072 (12)	−0.0110 (12)
C19	0.0329 (14)	0.0515 (17)	0.0379 (15)	0.0052 (13)	−0.0017 (12)	−0.0068 (13)
C20	0.0393 (16)	0.083 (2)	0.0410 (17)	0.0051 (17)	−0.0019 (14)	−0.0070 (17)
C21	0.050 (2)	0.111 (3)	0.0470 (19)	0.020 (2)	−0.0008 (17)	−0.028 (2)
C22	0.073 (3)	0.070 (3)	0.064 (2)	0.025 (2)	−0.009 (2)	−0.033 (2)
C23	0.057 (2)	0.0488 (19)	0.056 (2)	0.0120 (17)	−0.0086 (17)	−0.0192 (16)

### Geometric parameters (Å, º)

**Table:**

Br1—C20	1.898 (4)	C19—C20	1.376 (4)
O1—C1	1.234 (4)	C20—C21	1.387 (7)
O2—C15	1.190 (5)	C21—C22	1.357 (7)
O3—C16A	1.495 (8)	C22—C23	1.391 (5)
O3—C15	1.307 (4)	C2—H2B	0.9700
O3—C16B	1.47 (3)	C2—H2A	0.9700
O4—C13	1.216 (4)	C3—H3B	0.9700
O5—C23	1.361 (4)	C3—H3A	0.9700
N1—C5	1.391 (4)	C4—H4B	0.9700
N1—C9	1.392 (4)	C4—H4A	0.9700
N1—C14	1.461 (4)	C7—H7	0.9800
O5—H5	0.83 (3)	C10—H10A	0.9700
C1—C2	1.496 (5)	C10—H10B	0.9700
C1—C6	1.450 (4)	C11—H11A	0.9700
C2—C3	1.458 (6)	C11—H11B	0.9700
C3—C4	1.508 (5)	C12—H12A	0.9700
C4—C5	1.509 (4)	C12—H12B	0.9700
C5—C6	1.354 (4)	C14—H14A	0.9700
C6—C7	1.507 (4)	C14—H14B	0.9700
C7—C8	1.509 (4)	C16A—H16C	0.9700
C7—C18	1.524 (4)	C16A—H16B	0.9700
C8—C13	1.456 (4)	C16B—H16E	0.9700
C8—C9	1.347 (4)	C16B—H16D	0.9700
C9—C10	1.502 (4)	C17A—H17C	0.9600
C10—C11	1.499 (5)	C17A—H17A	0.9600
C11—C12	1.487 (4)	C17A—H17B	0.9600
C12—C13	1.501 (4)	C17B—H17D	0.9600
C14—C15	1.518 (5)	C17B—H17F	0.9600
C16A—C17A	1.462 (16)	C17B—H17E	0.9500
C16B—C17B	1.36 (6)	C19—H19	0.9300
C18—C23	1.403 (4)	C21—H21	0.9300
C18—C19	1.388 (4)	C22—H22	0.9300
			
C15—O3—C16A	113.9 (5)	C2—C3—H3A	109.00
C15—O3—C16B	129.4 (12)	C2—C3—H3B	109.00
C5—N1—C9	119.5 (2)	C3—C4—H4A	109.00
C5—N1—C14	119.0 (3)	C5—C4—H4B	109.00
C9—N1—C14	119.9 (2)	C3—C4—H4B	109.00
C23—O5—H5	110 (4)	H4A—C4—H4B	108.00
O1—C1—C6	120.8 (3)	C5—C4—H4A	109.00
C2—C1—C6	118.6 (3)	C6—C7—H7	107.00
O1—C1—C2	120.6 (3)	C8—C7—H7	107.00
C1—C2—C3	111.9 (3)	C18—C7—H7	107.00
C2—C3—C4	112.8 (3)	C9—C10—H10A	109.00
C3—C4—C5	111.6 (3)	C9—C10—H10B	109.00
C4—C5—C6	122.1 (3)	C11—C10—H10A	109.00
N1—C5—C6	120.9 (2)	H10A—C10—H10B	108.00
N1—C5—C4	117.0 (2)	C11—C10—H10B	109.00
C1—C6—C5	120.5 (3)	C10—C11—H11B	109.00
C5—C6—C7	122.0 (2)	C10—C11—H11A	109.00
C1—C6—C7	117.5 (2)	H11A—C11—H11B	108.00
C6—C7—C8	109.5 (2)	C12—C11—H11A	109.00
C6—C7—C18	113.0 (2)	C12—C11—H11B	109.00
C8—C7—C18	112.1 (2)	C13—C12—H12A	109.00
C9—C8—C13	120.7 (2)	C13—C12—H12B	109.00
C7—C8—C13	116.9 (2)	H12A—C12—H12B	108.00
C7—C8—C9	122.3 (2)	C11—C12—H12A	109.00
C8—C9—C10	121.7 (3)	C11—C12—H12B	109.00
N1—C9—C8	120.7 (2)	N1—C14—H14A	109.00
N1—C9—C10	117.5 (2)	C15—C14—H14A	109.00
C9—C10—C11	112.2 (3)	N1—C14—H14B	109.00
C10—C11—C12	111.5 (3)	H14A—C14—H14B	108.00
C11—C12—C13	111.8 (3)	C15—C14—H14B	109.00
O4—C13—C12	120.5 (3)	H16B—C16A—H16C	108.00
O4—C13—C8	121.3 (3)	O3—C16A—H16B	110.00
C8—C13—C12	118.2 (2)	C17A—C16A—H16C	110.00
N1—C14—C15	112.7 (2)	C17A—C16A—H16B	110.00
O3—C15—C14	110.8 (3)	O3—C16A—H16C	110.00
O2—C15—O3	124.6 (4)	C17B—C16B—H16D	112.00
O2—C15—C14	124.6 (3)	C17B—C16B—H16E	112.00
O3—C16A—C17A	108.1 (6)	O3—C16B—H16E	112.00
O3—C16B—C17B	100 (2)	O3—C16B—H16D	111.00
C7—C18—C23	121.2 (3)	H16D—C16B—H16E	110.00
C7—C18—C19	120.3 (2)	H17A—C17A—H17C	109.00
C19—C18—C23	118.5 (3)	H17B—C17A—H17C	110.00
C18—C19—C20	120.5 (3)	C16A—C17A—H17B	109.00
Br1—C20—C19	119.3 (3)	C16A—C17A—H17C	109.00
C19—C20—C21	120.9 (4)	C16A—C17A—H17A	110.00
Br1—C20—C21	119.8 (3)	H17A—C17A—H17B	109.00
C20—C21—C22	118.9 (3)	C16B—C17B—H17D	109.00
C21—C22—C23	121.6 (4)	C16B—C17B—H17E	110.00
C18—C23—C22	119.5 (3)	H17D—C17B—H17F	109.00
O5—C23—C18	121.9 (3)	H17E—C17B—H17F	110.00
O5—C23—C22	118.6 (3)	C16B—C17B—H17F	109.00
C3—C2—H2A	109.00	H17D—C17B—H17E	110.00
C3—C2—H2B	109.00	C20—C19—H19	120.00
H2A—C2—H2B	108.00	C18—C19—H19	120.00
C1—C2—H2B	109.00	C22—C21—H21	121.00
C1—C2—H2A	109.00	C20—C21—H21	121.00
C4—C3—H3B	109.00	C21—C22—H22	119.00
C4—C3—H3A	109.00	C23—C22—H22	119.00
H3A—C3—H3B	108.00		
			
C16A—O3—C15—O2	0.7 (6)	C18—C7—C8—C9	104.1 (3)
C16A—O3—C15—C14	179.5 (4)	C6—C7—C18—C23	−102.6 (3)
C15—O3—C16A—C17A	−163.9 (6)	C6—C7—C18—C19	79.3 (3)
C14—N1—C9—C8	177.7 (3)	C6—C7—C8—C9	−22.1 (3)
C9—N1—C14—C15	−81.1 (3)	C18—C7—C8—C13	−80.4 (3)
C14—N1—C5—C4	−1.8 (4)	C7—C8—C13—C12	179.1 (3)
C5—N1—C14—C15	84.6 (3)	C9—C8—C13—O4	172.3 (3)
C14—N1—C5—C6	179.7 (3)	C7—C8—C9—N1	7.6 (4)
C9—N1—C5—C4	164.0 (3)	C7—C8—C9—C10	−174.6 (3)
C5—N1—C9—C8	12.1 (4)	C7—C8—C13—O4	−3.3 (4)
C5—N1—C9—C10	−165.8 (3)	C13—C8—C9—N1	−167.8 (2)
C14—N1—C9—C10	−0.2 (4)	C9—C8—C13—C12	−5.2 (4)
C9—N1—C5—C6	−14.5 (4)	C13—C8—C9—C10	10.0 (4)
C2—C1—C6—C7	−173.2 (3)	C8—C9—C10—C11	17.9 (4)
C6—C1—C2—C3	−33.1 (5)	N1—C9—C10—C11	−164.2 (3)
O1—C1—C6—C7	5.4 (4)	C9—C10—C11—C12	−49.8 (4)
O1—C1—C6—C5	−175.2 (3)	C10—C11—C12—C13	54.3 (4)
O1—C1—C2—C3	148.3 (4)	C11—C12—C13—C8	−27.2 (4)
C2—C1—C6—C5	6.2 (5)	C11—C12—C13—O4	155.2 (3)
C1—C2—C3—C4	54.1 (5)	N1—C14—C15—O3	−175.9 (3)
C2—C3—C4—C5	−48.2 (5)	N1—C14—C15—O2	2.8 (5)
C3—C4—C5—N1	−157.2 (3)	C7—C18—C19—C20	176.5 (3)
C3—C4—C5—C6	21.3 (4)	C23—C18—C19—C20	−1.7 (4)
C4—C5—C6—C1	−0.6 (4)	C7—C18—C23—O5	3.0 (5)
C4—C5—C6—C7	178.8 (3)	C7—C18—C23—C22	−175.7 (3)
N1—C5—C6—C1	177.8 (3)	C19—C18—C23—O5	−178.8 (3)
N1—C5—C6—C7	−2.8 (4)	C19—C18—C23—C22	2.5 (5)
C5—C6—C7—C8	19.6 (4)	C18—C19—C20—Br1	−178.9 (2)
C1—C6—C7—C8	−160.9 (2)	C18—C19—C20—C21	−0.5 (5)
C5—C6—C7—C18	−106.0 (3)	Br1—C20—C21—C22	−179.7 (3)
C1—C6—C7—C18	73.4 (3)	C19—C20—C21—C22	1.9 (5)
C8—C7—C18—C19	−45.0 (3)	C20—C21—C22—C23	−1.1 (6)
C8—C7—C18—C23	133.2 (3)	C21—C22—C23—O5	−179.9 (4)
C6—C7—C8—C13	153.5 (2)	C21—C22—C23—C18	−1.1 (6)

### Hydrogen-bond geometry (Å, º)

**Table:**

*D*—H···*A*	*D*—H	H···*A*	*D*···*A*	*D*—H···*A*
O5—H5···O1	0.83 (3)	1.91 (3)	2.709 (4)	163 (6)
C7—H7···O5	0.98	2.47	2.917 (4)	107
C10—H10*A*···O4^i^	0.97	2.41	3.228 (4)	142
C14—H14*B*···O4^i^	0.97	2.42	3.267 (4)	146

Symmetry code: (i) −*x*+3/2, *y*+1/2, *z*.
