# Supplementary figures and images for: Crystal structure of ethyl 2-[9-(5-bromo-2-hy­droxy­phen­yl)-1,8-dioxo-1,2,3,4,5,6,7,8,9,10-deca­hydro­acridin-10-yl]acetate
Source: Acta Crystallogr E Crystallogr Commun. 2015 Nov 25;71(Pt 12):o989–90. doi: 10.1107/S2056989015022240 (PMC4719938; doi:10.1107/S2056989015022240)

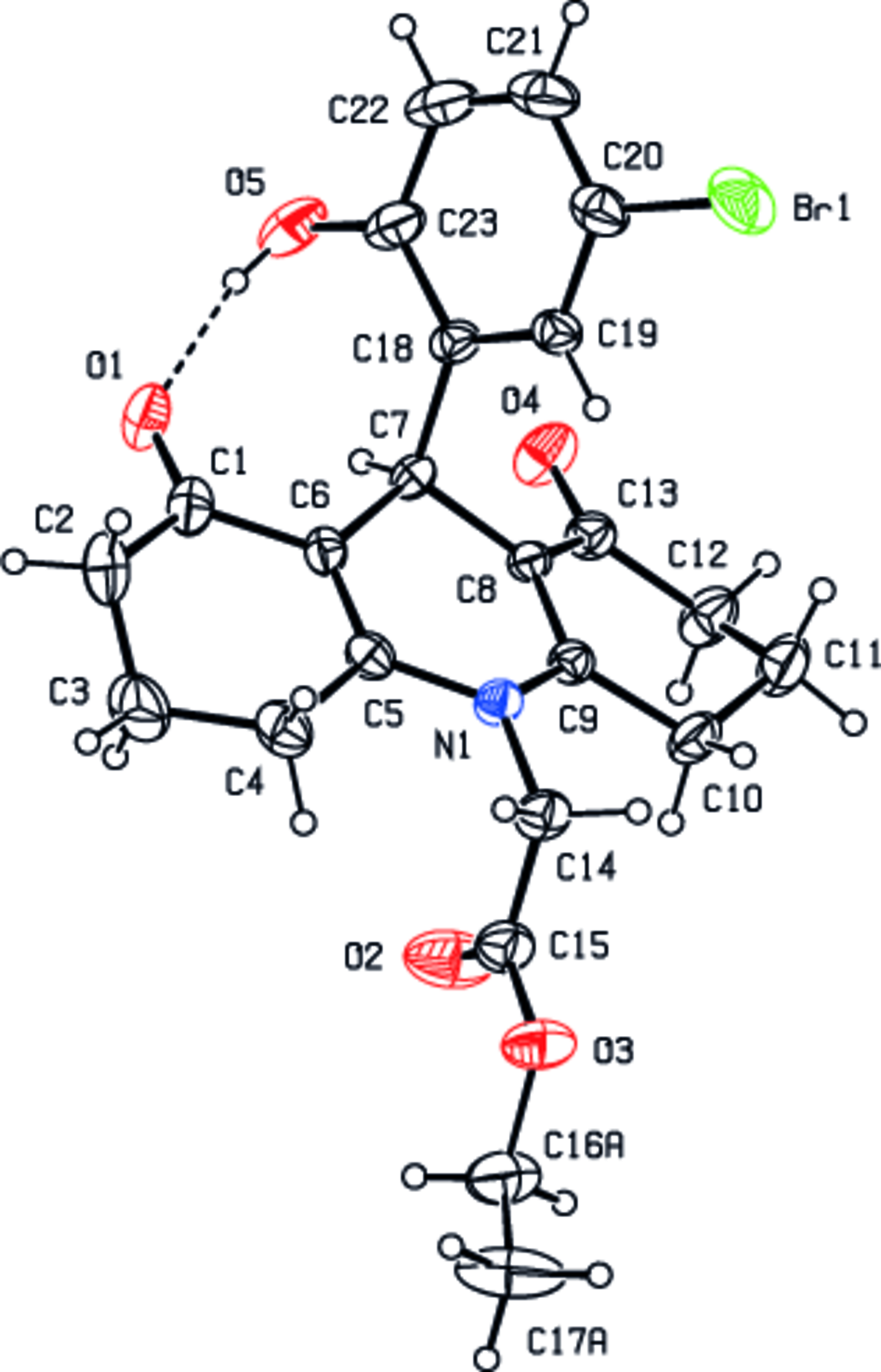

Supplement: Supplementary file 4 [file e-71-0o989-fig1.tif]

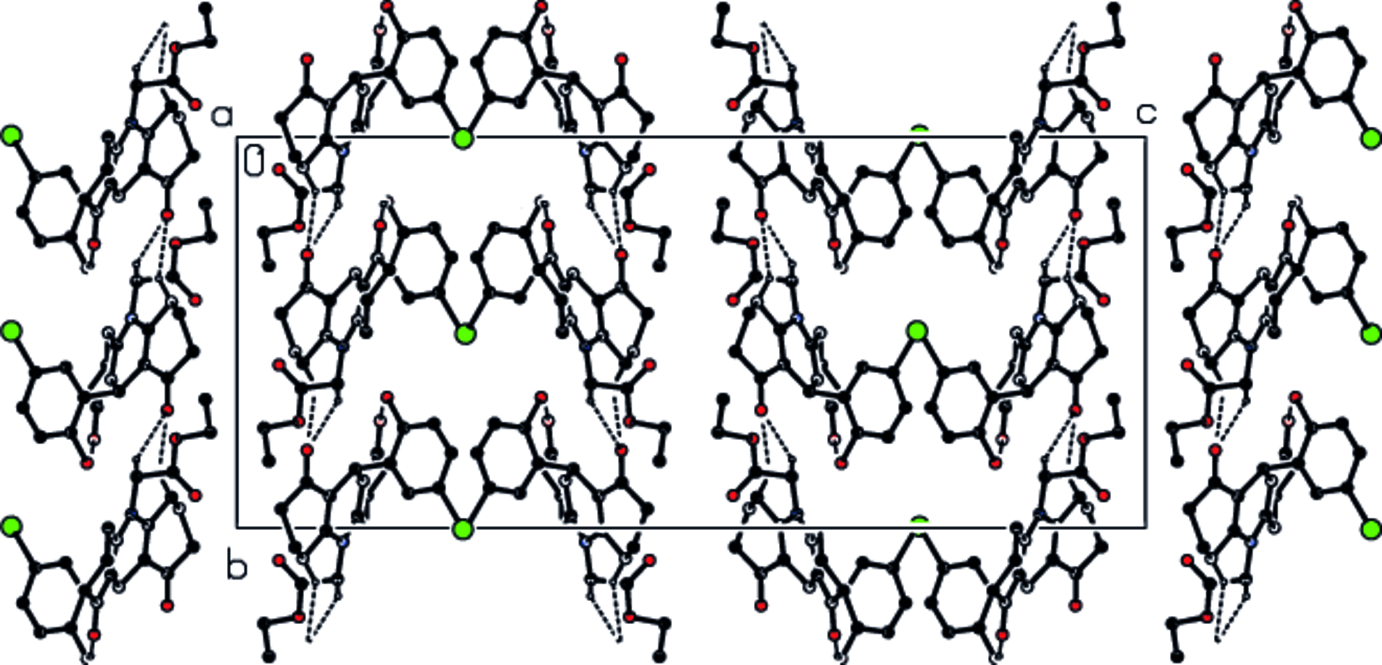

Supplement: Supplementary file 5 [file e-71-0o989-fig2.tif]
